# Statin use is associated with fewer periodontal lesions: A retrospective study

**DOI:** 10.1186/1472-6831-8-16

**Published:** 2008-05-15

**Authors:** Otso Lindy, Kimmo Suomalainen, Marja Mäkelä, Seppo Lindy

**Affiliations:** 1 Institute of Biomedicine/Biochemistry, University of Helsinki, Finland; 2 Institute of Dentistry, University of Helsinki, Finland; 3 University Dental Clinic, Health Centre of Helsinki, Finland

## Abstract

**Background:**

Inflammatory processes are considered to participate in the development of cardiovascular disease (CVD). Statins have been used successfully in the prevention and treatment of coronary heart disease. Chronic periodontitis has been suggested to contribute to CVD. The aim of this study was to examine the association of statin use and clinical markers of chronic periodontitis.

**Methods:**

Periodontal probing pocket depth (PPD) values were collected from dental records of 100 consecutive adult patients referred to a university dental clinic for treatment of advanced chronic periodontitis. A novel index, Periodontal Inflammatory Burden Index (PIBI), was derived from the PPD values to estimate systemic effects of periodontitis.

**Results:**

Periodontitis patients taking statins had a 37% lower number of pathological periodontal pockets than those without statin medication (P = 0.00043). PIBI, which combines and unifies the data on PPD, was 40% smaller in statin using patients than in patients without statin (P = 0.00069). PIBI of subjects on simvastatin and atorvastatin both differed significantly from patients without statin and were on the same level. The subjects' number of teeth had no effect on the results

**Conclusion:**

Patients on statin medication exhibit fewer signs of periodontal inflammatory injury than subjects without the statin regimen. PIBI provides a tool for monitoring inflammatory load of chronic periodontitis. The apparent beneficial effects of statins may in part be mediated by their pleiotropic anti-inflammatory effect on periodontal tissue.

## Background

Statins are inhibitors of hydroxymethylglutaryl coenzyme A (HMG-CoA) reductase. They were developed for the reduction of serum cholesterol levels [1], and have been used successfully in the prevention and treatment of coronary heart disease. [2-4]. Recently, interest has focused on non-cholesterol-dependent, pleiotropic effects of statins [5-7]. Anti-inflammatory pleiotropic effects are supposed to result from the inhibition of isoprene modification of signal transducers of inflammation [7].

Inflammatory processes are considered to participate in the development of cardiovascular disease (CVD) [8-11]. In addition to local inflammatory reactions in the arterial wall, chronic inflammatory processes in other tissues have been suggested to impose a systemic burden on the arteries, thus contributing to CVD. This systemic inflammation involves an increase in serum C-reactive protein, recently identified as a risk marker of atherosclerosis [12].

Gingivitis is one of the most prevalent localized inflammatory diseases in the adult population [13,14]. If left untreated, it may proceed to chronic periodontitis, a continuous inflammatory process resulting in irreversible periodontal tissue destruction [15]. This medium-grade inflammation may place a considerable burden on the cardiovascular system and contribute to CVD [15-18], and has been shown to be associated with systemic inflammation [19]. In this retrospective study, our aim was to examine the association of statin use and clinical indices of chronic periodontitis.

## Methods

The study protocol was approved by the Helsinki Health Centre Research Coordination Committee. Because the investigation involved only the review of patient records obtained during the course of medical care, no patient consent was required.

Data were collected from the dental records of 100 consecutive patients aged 40 years or older, who had been referred to the University Dental Clinic, Health Centre of Helsinki, for treatment of advanced chronic periodontitis during 2004–2005. Each patient was assigned an encrypted code linking the research data to the patient documents. Subjects with more than 14 teeth were included in the analysis. Patients using statins were compared with those who did not use statins.

We extracted the following data from the patient records: age, gender, reported current smoking, and use of statin medication. In addition, the records were checked for other medications, diabetes, rheumatoid diseases, and indicators of periodontal health.

A visible plaque index for index teeth [20] was extracted from the records.

For all teeth, we extracted recordings of six periodontal Probing Pocket Depth (PPD) values, measured to the nearest millimetre, from mesiobuccal, midbuccal, distobuccal, distolingual, midlingual, and mesiolingual surfaces using a WHO periodontal probe. [21-24]. Data on bleeding on probing was recorded for index teeth only and not included in the study. For a full dentition of 28 teeth, this yields 168 measurements. A gingival sulcus is considered physiological when PPD is less than 4 mm. The number of sites with moderate periodontal lesions (PPD at least 4 mm and less than 6 mm, modPPD) and advanced periodontal lesions (PPD 6 mm or deeper, advPPD) were recorded separately. For a dentition of 28 teeth, both of these indicators yield a maximum value of 6 × 28 = 168.

A novel index, Periodontal Inflammatory Burden Index or PIBI, was derived from the PPD values. The index is calculated by adding the number of periodontal sites indicating moderate periodontitis (N_modPPD_) to the weighted number of periodontal sites indicating advanced periodontitis (N_advPPD_).

(1) N_modPPD _= number of sites with moderate periodontal lesions

(2) N_advPPD _= number of sites with advanced periodontal lesions

(3) PIBI = ∑ (N_modPPD _+ 2 N_advPPD_)

If all measurement sites are advPPD in a 28-tooth dentition, PIBI can reach a maximum value of 336.

### Statistical analysis

Student's t-test was used to compare the means of the outcome variables following normal distribution. Kolmogorov-Smirnov test was used to estimate normality of the distribution, and Levene's test the equality of variances. The distribution-free Mann-Whitney U-test was applied to compare the medians when the values were not normally distributed [25]. The results are given as mean, standard error (SE), and the number of subjects in the group, unless otherwise indicated. The 95% confidence intervals and exact two-tailed p-values are given for each comparison.

In order to examine relationships between subjects' background variables and the number of periodontal pockets and PIBI, linear regression analysis was performed separately for the statin using patients and patients without statin.

Statistical calculations were performed using SPSS version 13.0 software (SPSS Inc., Chicago, Illinois) when appropriate.

## Results

### Patient characteristics

The demographic data of study subjects are presented in Table 1. Of the 100 consecutive patients referred to the clinic for periodontal treatment, three did not fulfil the inclusion criteria of having at least 50% of the dentition remaining. Of the eligible subjects (n = 97), 44 were women. The mean age of all study subjects was 53 years (range 42–69 years, Table 1).

**Table 1:** Description of study participants (n = 97)

	**Statin users**		**Non-users**	
	n	%	n	%
Patients	21		76	
Men	13	61.9	40	52.6
Women	8	38.1	36	47.4
Smokers	6	28.6	14	18.4
Diabetes	6	28.6	3	3.9
Rheumatic disease	1	4.8	5	6.6
				
	Years	Range	Years	Range
Mean age (SD)	57.4 (8.7)	44–69	52.3 (8.5)	42–69

The percentage of smokers was greater in statin users (28.6%) than in patients not receiving statin (18.4%). Diabetes was more frequent in statin users (28.6% vs. 3.9%). The percentage of patients with rheumatic disease was similar in both groups.

The number of remaining teeth of statin-using patients was 24.3 (SE 0.7) when that of non-using patients was 26.1 (SE 0.4). All subjects had poor oral hygiene, evident as visible dental plaque and gingival bleeding on probing.

Twenty-one subjects were on statin medication: 11 used atorvastatin, 7 simvastatin, 2 fluvastatin, and 1 rosuvastatin. All statin-using patients were receiving a conventional low-dose statin therapy.

### Periodontal inflammatory injury

**Figure 1:**
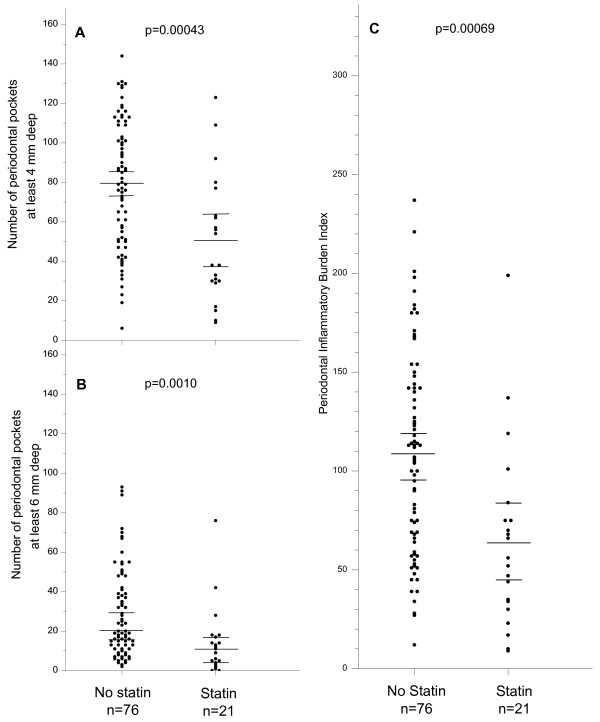
Scatter plots of the number of periodontal pockets and periodontal inflammatory burden index in subjects with and in subjects without statin medication. **A**. Number of periodontal pockets at least 4 mm deep. **B**. Number of periodontal pockets at least 6 mm deep. **C**. Periodontal inflammatory burden index. Long bars represent means in **A **and **C**, and medians in **B**. Short bars represent upper and lower 95% confidence limits.

Numbers of periodontal pockets in subjects with and without statin medication are shown in Figure 1 (means/medians and 95% CIs). In patients not using statins, the number of at least 4 mm deep periodontal pockets was 79.1 (SE 3.7; n = 76) (Figure 1A). In patients using statins, the corresponding number was 50.1 (SE 7.0; n = 21), being 37% lower than non-users. The difference of means was 29.0 (95% CI 12.9–45.0; P = 0.00043) (Table 2).

**Table 2:** Statin medication and periodontal inflammatory injury.

	Non-users n = 76	Statin users n = 21	Difference (% of control)	95% CI (difference of means)	P-value*
**A. Inflammatory burden**^†^					

PPD^‡ ^≥ 4 mm	79.1(3.7)	50.1 (7.0)	29.0 (-37%)	12.9 – 45.0	0.00043
4 mm ≤ PPD < 6 mm	50.7 (2.5)	36.0 (5.0)	14.7 (-29%)	3.2 – 26.2	0.0087
PPD ≥ 6 mm	20^§^	11^§^	11^|| ^(-55%)	4 – 20^¶^	0.0010**
PIBI^††^	107.6 (5.8)	64.3 (10.0)	43.1 (-40%)	19.5 – 66.7	0.00069

**B. Tooth- adjusted inflammatory injury **^‡‡^					

PPD ≥ 4 mm	3.1 (0.14)	2. 1 (0.30)	0.98 (-32%)	0.28 – 1.61	0.0025
4 mm ≤ PPD < 6 mm	2.0 (0.10)	1.5 (0.20)	0.48 (-25%)	0.02 – 0.93	0.0274
PPD ≥ 6 mm	0.7^§^	0.4^§^	0.42^|| ^(-56%)	0.14 – 0.76^¶^	0.0025**
PIBI	4.1 (0.22)	2.7 (0.44)	1.41 (-34%)	0.41 – 2.42	0.0033

* Two-tailed P-values. ^†^Number of periodontal pockets, mean (SE), unless otherwise indicated. ^‡^PPD is periodontal probing pocket depth. ^§^Median. ^||^Estimate for difference of population medians calculated as described by Campbell and Gardner [22]. ^¶^Approximate 95% confidence interval (CI) for the difference in population medians calculated as described by Campbell and Gardner [22]. **P-value by Mann-Whitney U-test. ^††^PIBI is Periodontal Inflammatory Burden Index. ^‡‡^Number of periodontal pockets divided by number of teeth, mean (SE), unless otherwise indicated.

Data were analysed separately for the number of moderate periodontal lesions and advanced periodontal lesions. In patients without statins, N_modPPD _indicating moderate periodontal lesions was 50.7 (SE 2.5; n = 76). In statin users, N_modPPD _was 36.0 (SE 5.0; n = 21), being 29% lower than in the non-using patients. The difference of means was 14.7 (95% CI 3.2–26.2; P = 0.0087) (Table 2).

Advanced periodontal inflammatory injury was measured as the number of periodontal pockets at least 6 mm deep. For patients without statin, N_advPPD _was 28.4 (SE 2.6; n = 76). The corresponding number for statin using patients was 14.2 (SE 3.8; n = 21), being 50% lower than in patients not using statins. The difference of means was 14.2 (95% CI 4.9–23.5; P = 0.0034). The N_advPPD _data indicating advanced periodontal lesions did not follow normal distribution. We therefore also used the distribution-free Mann-Whitney U-test to compare the population medians. The median was 20 for non-users and 11 for statin users. By the method of Campbell and Gardner [25], the estimated difference in population medians was 11 and the approximate 95% CI for the difference in population medians 4–20 (P = 0.0010) (Figure 1B, Table 2).

The Periodontal Inflammatory Burden Index (PIBI) combines and unifies data on PPD. In patients not taking statins PIBI was 107.6 (SE 5.8; n = 76) and in statin-using patients 64.3 (SE 10.0; n = 21) (Figure 1C). The difference of the means was 43.1 (95% CI 19.5–66.7; P = 0.00069). In statin users, PIBI was 40% smaller than in other periodontitis patients. For the non-smoking patients, PIBI was 64% lower for statin users when compared to patients without statin (48.9 vs. 105.2).

**Figure 2:**
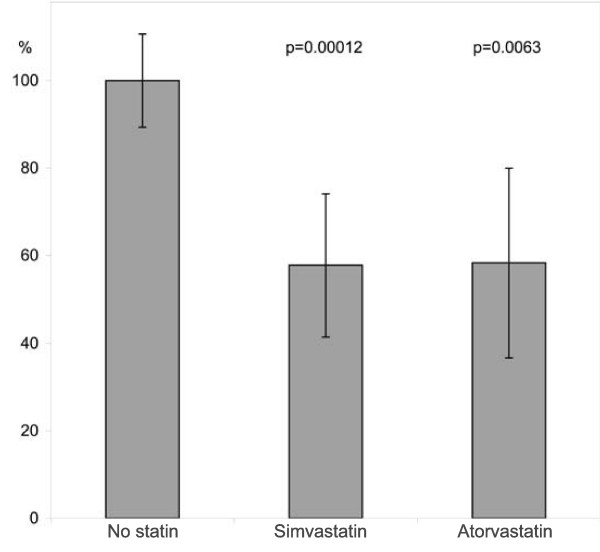
Statin subgroups and periodontal inflammatory injury. Comparison of periodontal inflammatory burden index. The columns represent non-using patients (set to 100%), simvastatin users and atorvastatin users. The whiskers represent 95% confidence intervals for the means.

The comparison of PIBI in subjects using simvastatin and atorvastatin is presented in Figure 2. Subjects on simvastatin as well as those on atorvastatin showed 40% lower mean PIBI values than subjects without statin. We observed no difference in PIBI between subjects on simvastatin or atorvastatin. The small number of subjects on fluvastatin or rosuvastatin did not allow their inclusion in the comparison.

To describe average periodontal injury, we divided the PPD values and PIBI by the number of teeth. The tooth-adjusted means of all of these clinical periodontal inflammatory signs were consistently lower in statin-users than in non-users (Table 2).

Linear regression analysis of the effect of background variables on the outcome variables revealed that smoking and diabetes were associated with a higher number of periodontal pockets among statin using patients, but not in those without statin (Table 3). The subject's age did not have an effect on the observed differences in the number of periodontal pockets between statin users and non-users. Age had no effect on PIBI in statin users, but explained 5% of the variation in PIBI of subjects not having a statin medication (Table 3).

**Table 3:** Linear regression of factors explaining periodontal injury and inflammatory burden measured as PIBI.

	**Patients without statin**				**Patients using statin**			
	PPD ≥ 4 mm		PIBI		PPD ≥ 4 mm		PIBI	
	r^2^	P	r^2^	P	r^2^	P	r^2^	P

gender	0.004	0.601	0.000	0.955	0.004	0.788	0.003	0.804
age	0.049	0.054	0.052	**0.047**	0.016	0.587	0.044	0.363
no of teeth	0.030	0.134	0.030	0.132	0.000	0.926	0.005	0.772
smoking	0.009	0.417	0.008	0.446	0.596	**4.2 × 10**^-7^	0.427	**0.001**
diabetes	0.002	0.729	0.006	0.511	0.203	**0.040**	0.250	**0.021**
RA	0.001	0.779	0.002	0.675	0.019	0.553	0.023	0.513

Squared regression coefficients (r^2^) and P-values (P) for baseline characteristics of periodontitis patients without statin and those receiving a statin. The values are shown for all pathological periodontal pockets (PPD ≥ 4 mm) and periodontal inflammatory burden index (PIBI). RA stands for rheumatoid arthritis.

## Discussion

### Strengths and limitations of the study

The primary data of this cross sectional, retrospective study based on clinical records of periodontitis patients can be considered reliable due to the extensive clinical examination protocol used for educational purposes in Finnish dental schools. The accuracy of the entries and the thoroughness of clinical examinations are controlled by senior staff members. The retrospective nature of the study places limits to external calibration of the measurements, and limits the interpretation of our measurements to the realm of a university dental clinic and the results are not necessarily applicable to a local dentist's practice.

Our subjects are Finnish adults from Helsinki area with advanced chronic periodontitis, either receiving regular statin medication or without statin medication. The number of teeth [26] as well as the percentage of statin users [27] in our subjects coincides well with that reported for this age group in Finland.

Clinical signs of periodontal tissue destruction have been reported to increase with age [28]. In our study this effect of age was evident for subjects not using statins. Interestingly, however, the effect of age could not be detected in statin users.

Diabetes is frequent among CVD patients [29]. This explains the higher percentage of diabetics in our statin-using patients when compared to non-users. Also, the proportion of current smokers was higher in statin-using than in non-using patients of this study. Both diabetes and smoking are predisposing factors for deterioration of periodontal tissue [15]. In spite of this, the positive effect on periodontal health observed in the statin using group was clear and statistically significant.

Low socio-economic status has been reported as a predisposing factor for chronic periodontitis [30]. CVD is associated with low socio-economic status [31]. Statin users are CVD risk patients. Thus, it is unlikely that statin medication selects patients with high socio-economic status and that this could affect the results.

The inflammatory potential of dentition is proportional to the number of teeth. All of our subjects had at least 15 remaining teeth, thus having at least 50% of the total possible dental inflammation sites. The small difference in the number of teeth between the groups did not affect the results. Statins had a substantial positive effect on local periodontal inflammatory injury, evident also when adjusted for the number of teeth.

### Periodontal inflammatory burden

Untreated periodontal pockets may serve as a source of persistent medium-grade inflammation with systemic consequences although the inflammation is milder and more local than in, for instance, rheumatoid arthritis. It is noteworthy that the prevalence of rheumatoid arthritis in the general population is about 1%, whereas the prevalence of gingival inflammation exceeds 50% [13]. Deep periodontal pockets liberate more inflammatory mediators and cytokines than shallow pockets, thus contributing more to the inflammatory burden. We used periodontal probing pocket depth (PPD) values to describe periodontal inflammatory injury. The number of tooth surfaces with increased PPD values reflects the total inflammatory burden caused by the local periodontal inflammation. The use of PPD instead of loss of clinical tooth attachment (PPD + gingival recession) is well reasoned in this context because measurements of clinical attachment loss do not reflect the inflammatory area of soft tissues surrounding the teeth.

The Periodontal Inflammatory Burden Index (PIBI) developed for this study takes into account the enlargement of inflamed subgingival surface area in the deeper periodontal pockets. This is achieved by weighting the number of advanced periodontal lesions with a coefficient 2 (see Equation 3). The actual value of the coefficient is probably greater than our estimate and remains to be elucidated in future studies.

### Periodontitis and CVD

Previous reports suggest that periodontal inflammation has systemic effects and can be a burden on remote tissues [19,32,33]. Chronic periodontitis causes deterioration of the diabetic condition [34], oral infections increase obstetric complications [35], and severe periodontitis is associated with systemic inflammation and a dysmetabolic status [19]. Reduced number of teeth has been shown to be a corollary of development of CVD [36,37] and an independent risk factor of aortic valve sclerosis [38]. The inflammatory component could be an important link between periodontal diseases and development of CVD [15-18,32,39,40]. Interestingly, although atorvastatin is a more potent cholesterol lowering compound than simvastatin [41], in our study both simvastatin and atorvastatin showed an equal association with low values of periodontal inflammatory burden index PIBI.

## Conclusion

Our results indicate that patients on statin medication exhibit fewer clinical signs of periodontal inflammatory injury than subjects without the statin regimen. This may lead to development of novel therapeutic approaches, if confirmed by consecutive prospective studies. In addition, further studies are needed to estimate the characteristics, value and applicability of PIBI in various clinical settings.

## Competing interests

The authors declare that they have no competing interests.

## Pre-publication history

The pre-publication history for this paper can be accessed here:


